# Facing the complex challenges of people with epidermolysis bullosa in Austria: a mixed methods study on burdens and helpful practices

**DOI:** 10.1186/s13023-024-03163-4

**Published:** 2024-05-21

**Authors:** Gudrun Salamon, Ursula Field-Werners, Sophie Strobl, Vinzenz Hübl, Anja Diem

**Affiliations:** 1 Faculty of Psychology, Sigmund Freud University Vienna, Freudplatz 1, 1020 Vienna, Austria; 2 EB House Austria, Department of Dermatology and Allergology, University Hospital of the Paracelsus Medical University, Salzburg, Austria

**Keywords:** Epidermolysis bullosa, Quality of life, Burden, Resources, Healthcare system

## Abstract

**Background:**

With approximately 500 people affected in Austria, epidermolysis bullosa (EB) is a rare genetic skin disease reducing the quality of life of those affected and their relatives. The intensive efforts of the patient organisation DEBRA Austria during the last decades have led to a unique situation of those affected and their relatives, with increased support and broader knowledge about the disease in the general population. The aim of the study is to evaluate the current situation of patients and their relatives living in Austria, with a focus on burdens and helpful practices.

**Results:**

The mixed-methods study consisted of two parts: a qualitative interview study to identify psychosocial aspects of EB in those affected and their relatives, and a subsequent online survey to further assess those aspects in a larger sample, resulting in a total of *n*=78 Austrian participants. The impact of EB on the quality of life of EB patients and their relatives in Austria is related to the current health status, psychological burden, mobility, visibility, financial situation as well as job prospects. Personal and social resources and external support have a significant influence on the individual situation.

**Conclusions:**

The outcome is mapped to concrete implications regarding targeted support for EB patients and their relatives on an individual level and their needs in regard to the Austrian health care system.

**Supplementary Information:**

The online version contains supplementary material available at 10.1186/s13023-024-03163-4.

## Introduction

Epidermolysis bullosa (EB) is a rare genetic disease group characterized by fragile skin. Even minimal trauma may lead to blisters and erosion, and daily wound care is time consuming and painful. The high diversity within the disease group is associated with major differences in regard to the affected body regions, the degree of severity, and the course of the disease. Symptoms range from localized blisters on the hands and feet, to those affecting the mucous membranes and gastrointestinal tract, to recurrent squamous cell carcinomas. Depending on the skin layer(s) affected, EB can be categorized into four types: EB simplex (EBS), junctional EB (JEB), dystrophic EB (DEB) and Kindler EB (KEB), which can again be differentiated into nearly thirty sub-types [1].

Despite intensive research, there is still no cure for EB, which is why medical treatment is primarily aimed at alleviating symptoms [2–4]. However, even with adequate medical support, the quality of life of people with EB and their relatives is usually seriously reduced [5–7]. Daily pain and itch, as well as wound care, take up a lot of time and significantly affect the daily routines of patients and their relatives [8, 9]. In addition, mobility as well as the ability to use the hands may also be limited [10, 11]. As can be easily imagined, the disease affects all aspects of life and leads to various psychosocial effects [12–15]. Not only does all of this impact the affected individuals themselves, but also their immediate environment, in particular their family and partner [16–19].

Worldwide, approximately 500.000 people have EB. In Austria, one in 17.000 children is born with EB, which leads to around 500 individuals and their families affected by EB. The situation for people with EB in Austria is somehow different to the one in any other country worldwide: Thanks to the intensive efforts of the EB patient organization DEBRA Austria^1^ during the previous twenty years, Austria has one of the most specialized EB treatment centres worldwide. The EB House Austria, a designated Centre of Expertise implemented in the European Reference Network for Rare and Undiagnosed Skin Diseases, combines an outpatient clinic with a clinical study centre, a research unit and the EB academy [20–24]. Moreover, DEBRA Austria's health policy interventions have resulted in larger parts of EB treatment being covered by the public health insurance, and due to the collection of donations, additional funding is available for further needs of EB patients and their families. Despite the rareness of the disease, because of regular media and billboard advertising campaigns, EB is familiar to a larger part of the Austrian population [23, 24]. However, the psychosocial intra- and inter-individual impact of EB on people with EB and their families and their situation in Austria has not been adequately studied. The aim of the current study is to identify the burdens, helpful practices and resources of Austrian EB patients and their families in dealing with this disease. The key results are translated into concrete public health strategies for improving quality of life with EB.

## Methods and materials

The study consisted of two parts: a qualitative interview study and a subsequent online survey. The aim of the interview study was to identify the psychosocial aspects of EB, whereas the online survey further explored those aspects in a larger sample. Study participants for both data collections were recruited in collaboration with the patient organisation DEBRA Austria and the EB House, with the former taking place from December 2019 to March 2020 and the latter from February to September 2021. Inclusion criteria for both studies were a diagnosis of EB or being a relative of a person diagnosed with EB, fluency in German, and a residence in Austria. The minimum age of the interview study was 10 years and of the online survey 14 years.

The qualitative face-to-face interviews were conducted in German by means of a semi-structured interview guide focusing on burdens and helpful practices for a life with EB. For participants under the age of 14, an adapted interview guide was used. Interviews ranged from 5 to 62 minutes, leading to a total of 474 minutes and 60,093 words analysed. The subsequent online survey was based on the identified relevant topics from the interviews. It consisted of sociodemographic questions, a series of standardized questionnaires and additional open-ended questions. The questionnaires elicited the current health status and related medical symptoms, assessed by the Instrument for Scoring Clinical Outcomes of Research for Epidermolysis Bullosa (iscorEB) [25–27], and the quality of life, assessed by the Quality of life in epidermolysis bullosa questionnaire (QOLEB) [28–31] for the patients and by the Epidermolysis Bullosa Family Burden of Disease (EB-BoD) [32] for the relatives. EB specific burdens, satisfaction, resources and helpful practices were assessed by the Resources for Life with an Illness for Epidermolysis Bullosa (ResILL-EB) questionnaire (Salamon G et al.: ResILL-EB, forthcoming), the social support assessed by the Perceived Social Support Questionnaire (F-SozU) [33, 34], the overall satisfaction assessed by the Satisfaction with Life Scale (SWL-5) [35, 36] and resilience assessed by the Brief Resilience Scale (BRS) [37–39]. Additionally, participants were asked four open questions to explore topics which were not addressed in the questionnaires regarding further burdens and helpful practices, additional support and stress.

Ethical approval was granted by the Medical and the Psychological Ethics Committees of the Sigmund Freud University Vienna. Informed written consent or assent was obtained by all participants or legal guardians of underage patients, respectively.

### Data analysis

Qualitative data from both data collection points were analysed using a literature-based structured coding system. In accordance to the recommendations given by the Thematic Analysis [40, 41], relevant topics were identified and merged to themes. Coding was carried out using MAXQDA software, version 2022. As for the quantitative data analysis, descriptive data testing was performed by the use of histograms, scatterplots, Kolmogorov-Smirnov tests, skewness and kurtosis statistics. The description of the study sample was based on the calculation of crosstabs, frequencies, percentages, means, medians, interquartile range (IQR) and standard deviations (SD in brackets). Due to the open online survey and the recruitment via stakeholders, no exact participation ratio can be calculated. Group comparisons were computed with non-parametric U-tests and Kruskal-Wallis tests, while point-biserial correlations were calculated to assess the relation between dichotomous and continuous variables. To counterbalance missing data and include as much collected data as possible, calculations for questionnaires were based on average scores instead of sum scores. All statistical calculations were performed using IBM SPSS Statistics software, version 27, and a significance level of 5% was assumed for entire analysis. Only significant results are reported. In order to include as many of the diverse aspects associated with EB as possible, the quantitative outcomes are combined with the qualitative results. Qualitative data was used to deepen and extend the quantitative results. The German interview passages presented in the paper were translated into English for comprehensibility reasons.

## Results

### Sample characteristics

An overview of the sample from the interview study and the online survey can be found in Table 1.

**Table 1:**
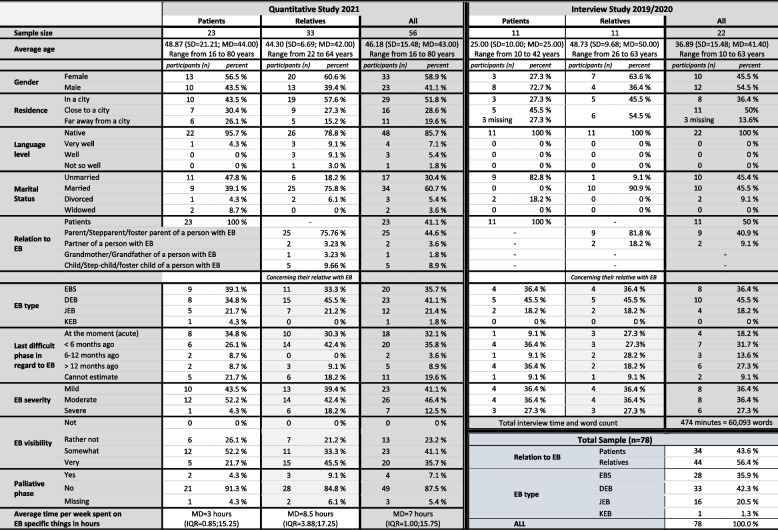
Sociodemographic characteristics of the patients and relatives

#### Interview study

In the first phase of the study project, *n*=22 interviews were conducted, 11 of which with EB patients and 11 with a relative or a partner of the patient. The group of the relatives consisted of 6 mothers, 3 fathers and 3 partners. The average age of the EB patients was 25 years (±10.00), ranging from 10 to 43, and of the relatives 48.73 (±9.68) years with a range of 26 to 63 years. With regard to gender, 45.5% were female and 54.5% male. Most of the relatives were married (90.9%), while the majority of the patients were unmarried (81.8%). With regard to the place of residence in Austria, 86.4% lived in or close to a city.

Concerning the EB types, 36.4% were diagnosed with EBS, 45.5% with DEB and 18.2% with JEB. The degree of severity was rated by an EB specialist as followed: 36.4% as mild, 36.4% as moderate and 27.3% as severe. At the time point of the interview, 9.1% of the patients indicated being in an acute EB phase, whereas 27.3% of the relatives stated that their child or partner was in an acute EB phase.

#### Online survey

The sample of the online survey consisted of *n*=56 participants, including 23 EB patients and 33 relatives of EB patients. The majority of the relatives were parents (17 mothers and 8 fathers) and the remaining were 2 children, 2 partners and 1 grandfather of an EB patient. The average age of the patients was 48.87 (±21.21) with a range of 16 to 80 years and of the relatives 44.30 (±9.69), ranging from 22 to 64 years. The median age of the EB relatives of the relatives was 10, ranging from 1 to 56 years. In both subsamples, slightly more women participated in the survey (56.5% and 60.6%, respectively). With regard to the marital status of the participants, nearly half of the patients were unmarried (47.8%), whereas 75.8% of the relatives were married. In both subsamples, the majority was in a relationship (73.9% and 90.9%, respectively). Nearly all of the patients (95.7%) were German native speakers and 78.8% of the relatives. Regarding the place of residence in Austria, most of the patients and relatives lived either in a city (43.5% and 57.6%, respectively) or close to a city (30.4% and 27.3%, respectively).

With regard to the distribution of the four major EB types in both groups, slightly more patients were diagnosed with EBS (39.1%) than with DEB (34.8%), 21.7% with JEB and 4.3% with KEB. In contrast, a higher percentage of patients whose family member completed the survey had a diagnosis of DEB (45.5%), followed by EBS (33.3%) and JEB (21.2%). Concerning the degree of severity, most of the patients rated their EB as mild (43.5%) or as moderate (52.2%), similar ratings were obtained from the relatives with 39.4% as mild and 42.4% as moderate. All of the participants stated that their EB or the EB of their family member was visible to a certain degree. At the time point of the survey, around one third of the EB patients in both samples were in an acute EB phase (34.8% and 30.3%, respectively) and four patients in a palliative phase. In both groups, the most commonly affected body parts of the EB patients were the feet (87.5%), legs (67.9%) and hands (64.3%), which caused restrictions in their mobility in more than half of the patients. Due to these mobility limitations, one patient had to use a grip aid and ten patients a walking aid or wheelchair.

### Aspects influencing life with EB in Austria

In order to capture the full diversity of living conditions with EB, the subsequent calculations were performed for the subgroups of patients and relatives separately and thereafter for the combined dataset. To be able to cover the broad spectrum of psychosocial effects of EB in this paper, only significant results are presented. A group comparison matrix of all categories’ medians can be found in the additional material (Table A1).

With regard to the **current health status **of the EB patients in both subsamples of the survey, patients who were diagnosed with JEB and DEB had significantly more medical symptoms than those with EBS (H(2)=16.79, *p*<.001). Furthermore, patients with moderate to severe EB as well as with a very visible EB had significantly more medical symptoms (iscorEB) that those with a mild and hardly visible EB (severity: U=589.50, z=4.62, *p*<.001 and visibility: H(2)=6.75, *p*=.034). Patients who were in an acute ER phase at the time point of the survey had significantly more medical symptoms than those who were not (U=340.00, z=2.84, *p*=.005). Additionally, female patients reported more medical symptoms than male (U=18.00, z=-2.47, *p*=.014).

Patients and relatives of EB patients with **limitations in their mobility** reported significantly more medical symptoms than those who were not restricted (iscorEB: U=493.00, z=2.75, *p*=.006). Furthermore, these patients and relatives had fewer available resources than those who were not limited in their mobility (ResILL-EB Resources scale: U=163.50, z=-2.00, *p*=.046).

With regard to **burdens**, there were significant differences on the ResILL-EB Burden scale concerning the degree of severity, visibility and EB phase. The patients whose EB was rated by themselves or their family members as moderate to severe or as very visible were more burdened than the mild or hardly visible ones (severity: U=464.00, z=3.25, *p*=.001 and visibility: H(2)=8.99, *p*=.011). Furthermore, patients or relatives who reported an acute EB phase scored significantly higher on the ResILL-EB Burden scale (U=281.00, z=2.46, *p*=.014). Patients who were diagnosed with JEB and DEB scored significantly higher on the ResILL-EB Burden scale than those with EBS (H(2)=10.92, *p*=.004). With regard to the patient group, there were significant differences between female and male survey participants. Women reported more burdens than men (U=19.00, z=-2.24, *p*=.025).

Quality of life of the patients and the relatives seems to differ according to severity and current acute phase. With regard to the QOLEB, patients who rated their EB as moderate to severe or who were in an acute EB phase had a lower quality of life than those with mild EB or who were not in an acute phase (severity: U=83.50, z=2.29, *p*=.022; acute phase: U=59.50, z=2.27, *p*=.023). On the EB-BoD, relatives who rated their family member’s EB as moderate to severe were more burdened than those who rated it as mild (U=140.50, z=2.79, *p*=.005). On the QOLEB, female patients scored significantly higher than male patients (U=22.00, z=-2.18, *p*=.029), on the EB-BoD, lower scores were observed in female relatives than in men (U=141.50, z=2.65, *p*=.008). This indicates that in regard to quality of life, female patients and male relatives feel more burdened.

The **financial burden** of the participants was a common topic emerging from the interviews. Participants who were more burdened by their financial situation due to EB had more medical symptoms, less social support, lower satisfaction with life and fewer resources available than those who were rather not or not burdened. Significant differences were found on the iscorEB: U=446.50, z=3.76, *p*<.001, the F-SozU: U=117.00, z=-2.60, *p*=.009, the ResILL-EB Satisfaction scale: U=149.00, z=-2.45, *p*=.014, the SWLS: U=91.50, z=-3.22, *p*=.001 and the ResILL-EB Resources scale: U=66.00, z=-4.04, p<.001. For relatives, a higher financial burden was associated with a lower quality of life (EB-BoD: U=149.00, z=4.11, *p*<.001). Regarding the satisfaction with their financial situation, again, participants who were less satisfied had more medical symptoms, were more burdened, had less social support, were less satisfied with their life and had fewer resources. Significant differences were found on the iscorEB: U=106.50, z=-3.57, *p*<.001, the ResILL-EB Burden scale: U=140.00, z=-2.85, *p*=.004, the F-SozU: U=310.00, z=2.51, *p*=.012, the SWLS: U=324.50, z=2.88, *p*=.004 and the ResILL-EB Resources scale: U=385.50, z=3.10, *p*=.002. Patients who were less satisfied with their financial situation, stated that their quality of life was lower (QOLEB: U=18.50, z=-2.36,* p*=.018). On top of the financial burden, patients and relatives often have to go through various bureaucratic procedures to obtain financial support.

“You always have to make sure you get time off. For me it was always more of a fight with the BVA [Austrian health insurance], I have to say. The authorities were the bigger problem, almost bigger than the disease itself. We always had to go there and say we’d appeal, again and again, because they just kept rejecting it.” (A3, pos. 2)

The struggle to **find a job** which fits the EB patients’ needs and wishes was frequently articulated by the interviewees. In opposition to the relatives who regarded their career more as a financial necessity, which some would willingly give up to care for their loved ones, the EB-patients’ perception of a professional life was more focused on self-fulfilment. For some, a professional career was connected to the idea of a meaningful life. For many people with EB, it is not easy to find a suitable job. A sheltered workplace may be a good solution for some, but others describe the struggles of working in a sheltered workplace, which may fit their physical but not their cognitive or social needs.

“Opportunities to work in facilities that employ physically and not only multiply disabled people. The existing facilities such as 'Lebenshilfe' or sheltered workshops are not suitable for people with EB because they are only physically but not mentally impaired.” (860, pos. 2)

“I don't know why it's so difficult. For people who have an impairment, they are told right from the start that there is no place for you. I do believe that there would be places for them.” (A10, pos. 3)

Moreover, if EB patients manage to find a job, they often lose the financial support they still need for wound care and dressings. As a result, they are faced with an even greater financial burden and feel unjustly punished for their willingness to work.

“Now they [the authorities] say that he has a job now and wants to go to work, that's fine, but they just say that if he can go to work, he doesn't need care anymore. What do you say to that as a parent? You have no choice but to acknowledge that. The older one gets, the more everything gets cut.” (A3, pos. 2)

“The legal situation in Austria […] discriminates against people with EB for receiving an occupational disability pension if they take up a job despite their disability and later actually become unable to work.” (860, pos. 2)

Especially for parents of children with EB, the overwhelming amount of different tasks can lead to overload and to **psychological burden**. In this regard, the participants pointed out the incompatibility of a high effort family-life, which includes day-to-day care and organisational tasks, with the mentioned need to stay financially solvent. In addition, it is sometimes difficult for relatives to delegate tasks because externals, even health care professionals, do not know enough or lack the years of experience in dealing with this individual EB patient.

“I am usually not overburdened with EB alone – it is always the sum of all the duties, besides work, household and EB care – without relatives nearby and without a babysitter, without a defined time-out alone or, even more rarely, with my husband. Then it happens that I snap at my other family members and treat them unfairly because I feel ‘left alone’ and responsible for everything.“ (627, pos. 3)

“Honestly, it's pretty much stuck with me, I have to say, the [care] work and all that. Everybody is like, yeah, if you need something and stuff. But in reality, people are very scared, although you don't have to be scared, you can learn all that, how to deal with a person like that.” (A10, pos. 2)

The correlation matrix of the scores in Table 2 shows how the scales are interrelated. Thereby, it becomes apparent that poorer health is associated with higher EB burden, poorer quality of life, fewer resources, less social support and lower satisfaction (EB specific and overall). Similarly, poorer quality of life correlates significantly with a higher burden, poorer satisfaction and fewer resources, with a strong effect for each correlation. While relatives with poorer quality of life also indicate to have significantly lower social support, there is no significant correlation in this regard in EB patients. In addition, people with a higher EB burden have poorer satisfaction (EB specific and overall), fewer resources, less social support, and report lower resilience. In contrast, participants with higher social support, more resources and more helpful strategies tend to have a significant higher satisfaction. Those with more resources show higher resilience and satisfaction and indicate to have more helpful practices and social support available.

**Table 2:**
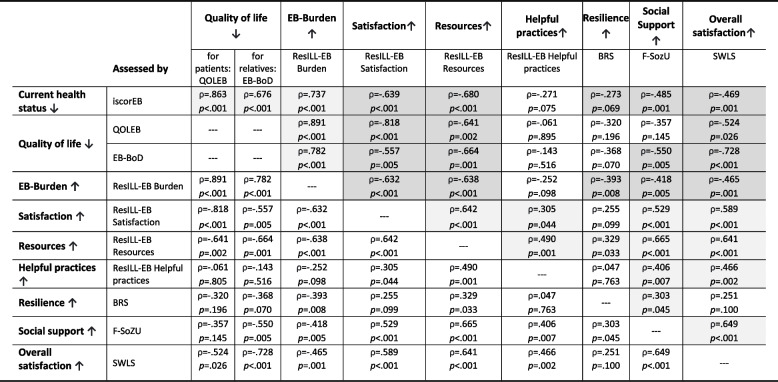
Cross-correlation matrix of the questionnaires

↑ = Higher values indicate higher expression in the targeted construct; ↓ = Higher values indicate lower expression in the targeted construct

#### Resources, support and contentment

With regard to **social and personal resources**, significant differences were found in relation to the degree of severity, EB phases and EB types. Concerning severity and acute EB phase, EB patients and relatives of EB patients whose EB was rated as mild or who were not in an acute EB phase were more satisfied as measured by the ResILL-EB Satisfaction scale (severity: U=169.50, z=-2.24, *p*=.025 and acute phase: U=62.50, z=-3.47, *p*=.001). Furthermore, patients and relatives of patients who were not in an acute EB phase had more resources available (ResILL-EB Resources scale: U=104.00, z=-2.14, *p*=.033) and were more resilient (BRS: U=89.00, z=-2.13, *p*=.033). Relatives indicated to have significantly more social support available if their family member with EB was not in an acute phase (F-SozU: U=20.00, z=-2.17, *p*=.030). On the ResILL-EB Satisfaction scale, patients and relatives of patients who were diagnosed with EBS were more satisfied with their overall EB situation than those with DEB and JEB (H(2)=14.65, *p*=.001). Similar results were also found on the SWLS, with a higher life satisfaction of EBS patients and their relatives (H(2)=6.15, *p*=.046). Furthermore, EBS patients or relatives of EBS patients scored significantly higher on the ResILL-EB Resources scale (H(2)=10.31, *p*=.006), indicating that they had more resources available than the remaining patients or relatives. Differences between female and male participants were found concerning perceived social support and the amount of helpful practices available for dealing with EB. Women indicated to have significantly more support available (F-SozU: U=120.00, z=-2.80, *p*=.005) and scored significantly higher on the ResILL-EB Helpful practices scale (U=126.50, z=-2.64, *p*=.008) than men. With regard to the subsample of the relatives, women had more resources available (ResILL-EB Resources scale: U=16.00, z=-3.38, *p*=.001) and were more satisfied with life (SWLS: U=44.00, z=-2.01, *p*=.045) than men.

With regard to the **psychological impact** of EB on the participants, there were significant differences between happy and unhappy participants. The participants who were in a happy mood at the time point of the survey reported fewer burdens and a higher quality of life, had more social support, were more satisfied with their situation, and had more resources and more helpful practices available for dealing with EB. Significant differences were found on the ResILL-EB Burden scale: U=442.50, z=2.71, *p*=.007, the QOLEB (patients): U=94.30, z=2.79, *p*=.005, the EB-BoD (relatives): U=128.50, z=2.00, *p*=.045, the F-SoZU: U=150.00, z=-2.08, *p*=.037, the ResILL-EB Satisfaction scale: U=129.00, z=-3.16,* p*=.002, the ResILL-EB Resources scale: U=141.00, z=-2.64, *p*=.008 and the ResILL-EB Helpful practices scale: U=147.50, z=-2.07, *p*=.038. Participants who reported to be burdened by their feelings had significantly more medical symptoms, were less satisfied and had fewer resources available (iscorEB: U=442.00, z=2.87,* p*=.004; ResILL-EB Satisfaction scale: U=151.00, z=-2.80, *p*=.005; ResILL-EB Resources scale: U=168.00, z=-2.04, *p*=.041). Additionally, patients who were burdened by their feelings, reported a lower quality of life (QOLEB: U=80.50, z=2.78, *p*=.005). Concerning the feelings of worries and fears, significant differences were found on all questionnaires (iscorEB: U=515.50, z=4.32, *p*<.001; QOLEB (patients): U=89.00, z=3.17, *p*=.002; EB-BoD (relatives): U=137.00, z=3.29, *p*=.001; ResILL-EB Satisfaction scale: U=108.00, z=-3.71, *p*<.001; SWLS: U=112.00, z=-3.05, *p*=.002; ResILL-EB Resources scale: U=71.00, z=-4.23, *p*<.001; ResILL-EB Helpful practices scale: U=129.00, z=-2.63,* p*=.009; BRS: U=141.00, z=-2.38, *p*=.017; F-SozU:U=151.00, z=-2.14, *p*=.033). Hence, participants who were more burdened by their worries and fears reported more medical symptoms, a lower quality of life, less satisfaction, fewer resources and helpful practices as well as less resilience and social support.

Concerning the distribution of **care within the family system**, participants reported a variety of strategies. While most families were dividing the responsibilities in a more traditional way, some distributed the care workload equally. In families with mothers as the primary care givers, fathers in turn often provided emotional, financial and/or organisational support for their partners. Nevertheless, families or family members often faced times of overwhelming stress. To reduce or even prevent too much stress, participants reported that explicit parental care agreements and the negotiation of “terms and conditions” were helpful. In this regard, consultation of a professional counsellor or psychologist was experienced as beneficial.

“My wife was quite distressed, I think she was on the verge of burnout, and she refused to get psychological help, and finally she did, but it wasn't a psychologist, it was a very nice person who […] did some kind of coaching, and she came actually only once […] and she had a long conversation with my wife and then she changed some things. She had told her, for example, […] you can't get away from the fact that your daughter is attached to you and the daughter actually only wants to be taken care of by you, but you have to give up something else, you can't do everything, do household chores, cleaning, cooking, washing, ironing and then looking after the daughter, then it becomes too much. My wife realized that, and we now have a cleaning lady who comes once a week and makes sure that everything is in order here.” (A6, pos. 41)

Concerning care, professional support has been repeatedly described as highly beneficial. Several participants reported that their children with EB benefit from having multiple caregivers, e.g., by including professional caregivers. The early involvement of multiple caregivers can reduce the risk of the EB patient becoming emotionally and socially dependent on only one person. It also increases the flexibility of both, the EB patient and the main caregiver. In this context, relatives retrospectively reported that it would have been helpful to include several caregivers in wound management from early on.

“Unfortunately, we overlooked the idea of getting a nurse or an external person to take care of her when she was a toddler.” (A6, pos. 2)

“It was also mentally demanding. We did a division of duties, good cop, bad cop, when it came to wound care. We had the privilege of always doing it as a trio. [… My wife] was responsible for the psychological care, so to speak for being there, and I was just the bad guy. On the one hand, that welded us together as a care community. But at the same time, there were often such extreme situations where I, in particular, really really shut down emotionally and really wanted to have my peace. That didn't necessarily bring us together as a relationship. Those were very difficult years, and some of them still are.” (A9, Pos. 2)

**Fig. 1:**
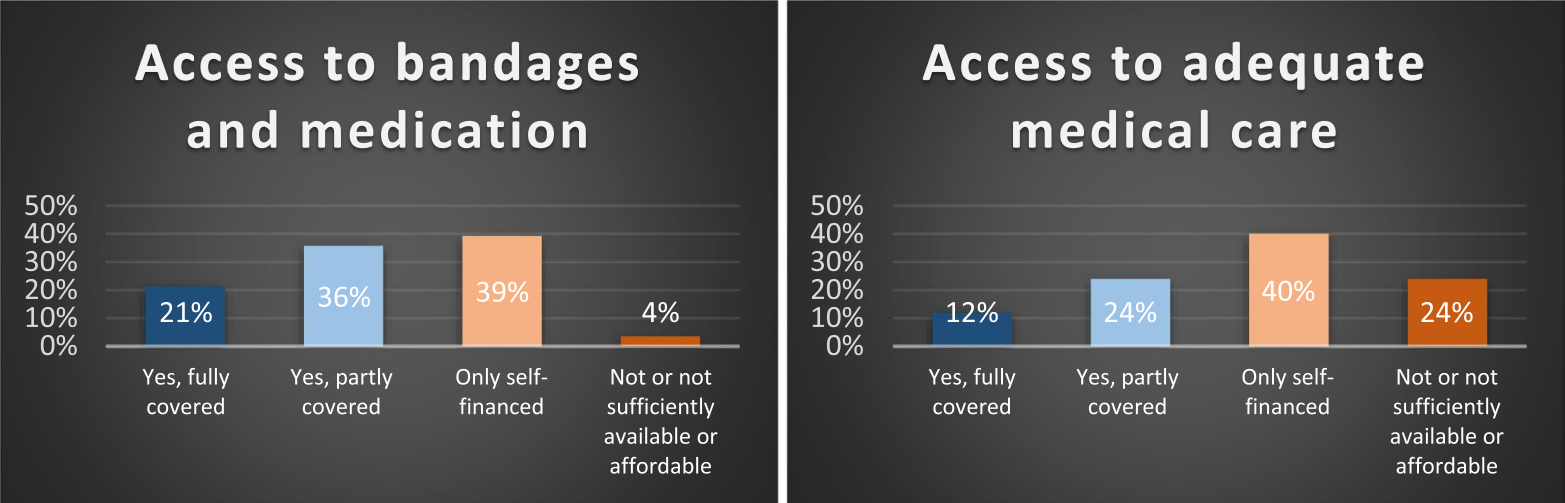
Access to bandages, medication and to adequate medical care

In terms of **financial support**, more than half of the participants reported that the costs of bandages and medication were fully or partially covered, but only a third had full or partial access to adequate medical care. However, only 21% and 12%, respectively, reported to have all their related expenses covered, whereas 40% of the participants had to fully fund their own bandages and medication and adequate medical care. While only 4% reported that they did not have adequate access to bandages and medication due to a lack of funds and/or availability, this was the case for 24% in regards to adequate medical care (see Fig. 1).

**Fig. 2:**
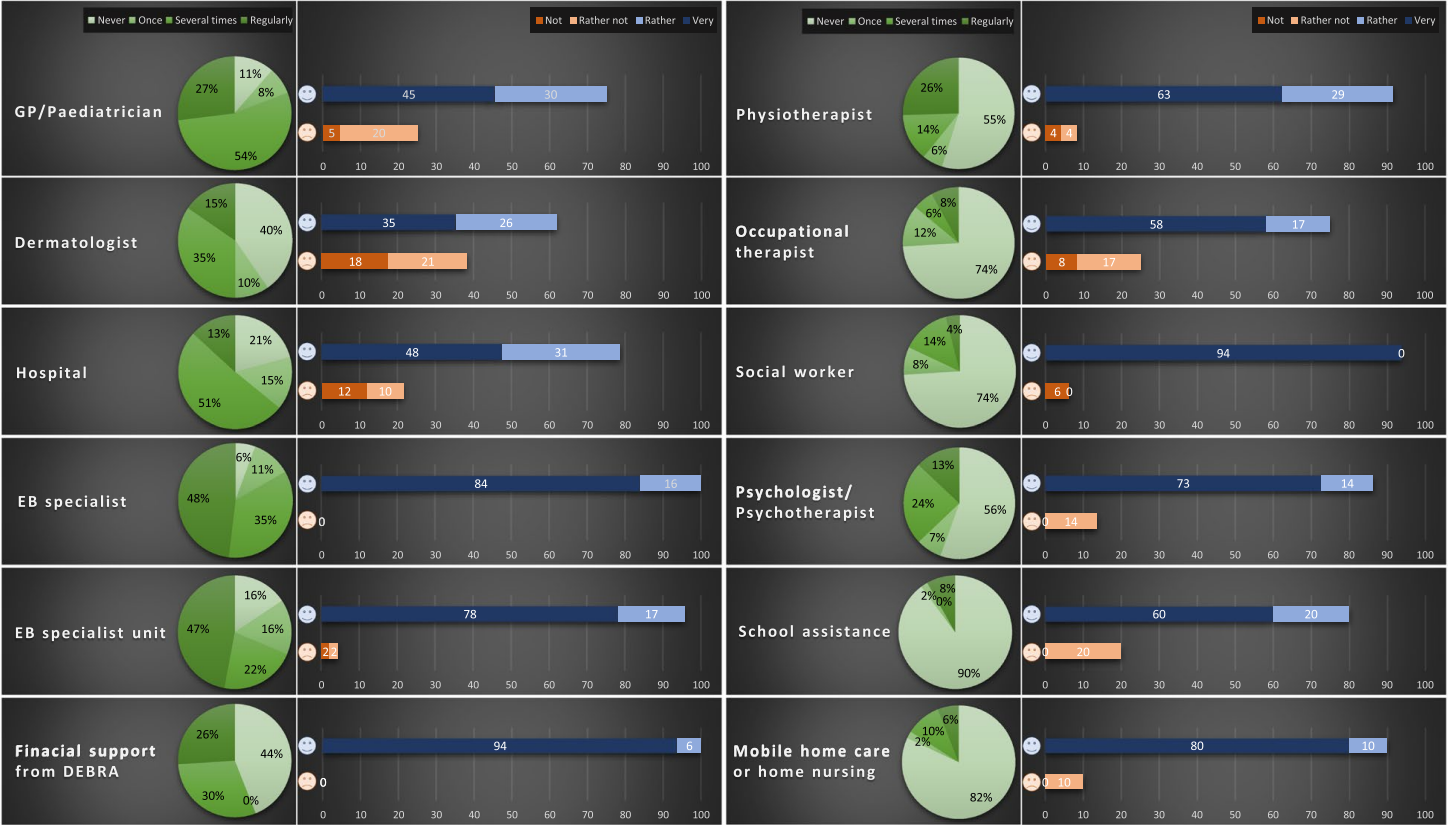
Frequency of use of medical service providers or institutions and contentment

With regard to the **external support**, we assessed the frequency of use of the support given by the medical service providers or institutions as well as the contentment with them (see Fig. 2). More than half of the participants used medical facilities, i.e., general practitioner (GP), dermatologist, hospital and EB specialist (GP, dermatologist or nurse working in the EB specialist unit), several times to regularly. The overall contentment with the EB specialist was 100% and 95.7% with the EB specialist unit. In contrast, the majority of participants had never or only once sought support from other services such as occupational therapists, social workers, school assistance and mobile home care. Despite the lower frequency of use, the contentment with these services was high. With regard to the area of psychological and psychotherapeutic support, 37% of the participants had frequent to regular support. Around half of the participants (56%) had received financial support from DEBRA Austria several times to regularly and were 100% content with this support. Financial support is provided upon request and aims to ensure appropriate care for EB families, e.g., by helping to cover the expenses for bandages, medication or qualified healthcare professionals.

Thereafter, the overall support and contentment was calculated. Around one third of the participants received advice and support regularly (30.9%) and the vast majority (90.9%) was very content with the services (see Fig. 2).

Participants who reported more medical symptoms and who were more burdened had more frequent and regular contact with the diverse service providers or institutions. High frequency of overall support was associated with more medical symptoms (iscorEB: *r*_*pb*_=0.52, *p*<.001) as well as with more burdens (ResILL-EB Burden scale: *r*_*pb*_=0.35, *p*=.015). There were significant differences between participants who did and who did not receive frequent support concerning medical symptoms (iscorEB: U=464.50, z=3.51, *p*<.001) and burdens (ResILL-EB Burden scale: U=361.00, z=2.30, *p*=.021). Those participants who frequently and regularly sought psychological or psychotherapeutic support reported more medical symptoms and more burdens (iscorEB: U=463.50, z=2.97, *p*=.003; ResILL-EB Burden scale: U=375.50, z=2.11, *p*=.035), but also had more helpful practices available than those who did not seek support (ResILL-EB Helpful practices scale: U=274.00, z=2.09, *p*=.037).

The ratio of high and low service use frequency and contentment was calculated. Participants who frequently used the services were mainly content (73.77%) and hardly discontent (9.26%) with them. Among the small percentage of participants who used the services with low frequency, the overall contentment was four times higher than discontentment (Table 3).

**Table 3:**
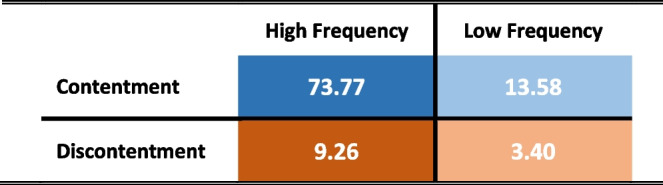
Frequency of overall support use and contentment (in percentages)

## Discussion

The aim of the reported mixed method study was to evaluate the psychosocial effects of EB on patients and their relatives living in Austria, with a focus on burdens and resources.

With regard to the prevalence of EB in Austria of around 500 people, the sample sizes of both the qualitative and the quantitative studies can be considered as highly representative. In both the interview study as well in the online survey, the majority of the relatives were parents although all relatives were invited to participate. This could be due to the fact that parents are the relatives most involved in EB care [42]. The distribution of EB types in both samples is similar to the distribution in clinical practice [43], with EBS and DEB being more common than JEB and Kindler, the latter being the rarest EB type. Many differences between the three major EB types were found concerning the current health status, burdens, satisfaction with their situation and life and resources. People with EBS tended to report fewer medical symptoms, less stress, more satisfaction and more resources than people with DEB and JEB. This is in accordance with EBS being described as the type with the relatively mildest clinical pictures of epidermolysis bullosa with an immense, but slightly lower and also different impact on quality of life [5, 44, 45]. Differences between EB subtypes could not be calculated as most participants did not report the specific EB subtype. As symptoms can vary greatly between subtypes, future studies should take these into account.

With the help of an adapted interview guide for younger participants, an age limit of 10 years could be chosen for the interviews, whereas a minimum of 14 years was chosen for the online survey. However, in the quantitative study, information about younger EB patients were included through reports of their relatives. Despite the wider age range in the online survey patient group, there is no bias as the subgroups are relatively similar in terms of mean age and medians.

Concerning the gender distribution, the ratio of female and male participants was nearly equal, which permitted the analysis of gender differences. Women had more social support and more helpful practices available for dealing with EB. These results are in accordance with previous studies that women tend to seek more social support and are more likely to use a wider range of coping strategies than men [46]. However, in the patient subsample, quality of life results were significantly lower for female participants, with women reporting more medical symptoms and more burden. In contrast, males indicated a lower overall quality of life among the group of relatives. Female relatives reported more resources, which come along with a higher satisfaction and higher overall quality of life. As to the observed gender differences in the subgroups of patients and relatives, the sample sizes of both groups were quite small, partly due to some missing data. Even though previous findings describe female relatives as more burdened [17, 47], it would make sense to further investigate the different burden experienced by male and female relatives in order to develop targeted support for both.

In the online survey, patients and relatives rated the degree of severity and visibility of their or their relative’s EB. The inclusion of these subjective measures was based on the assumption that patients themselves as well as their relatives are experts of their own health or the health of a person close to them. Moderate to severely affected EB patients had more medical symptoms. Furthermore, the degree of severity affected them and their relatives so that they were more burdened, less satisfied with their situation as well as reported a lower quality of life. Concerning the severity of EB, the majority of patients rated their EB as mild or moderate and only one as severe. In contrast, more severe cases were reported in the group of relatives, which indicates that severely affected EB patients are less likely to take part in a survey due to their overall health. Due to the lower number of severe cases, especially in the patient group, for the group comparisons, the cases of moderate and severe were added together. Furthermore, our results showed that the patients and relatives of patients whose EB was very visible had more medical symptoms and felt more burdened. The latter can be explained by the higher amount of medical symptoms, but might also be influenced by the various stigmata associated with the visibility of the disease, which can lead to unpleasant comments, stares or even discrimination and bullying [15, 48]. The results of this paper emphasise that additional to the evaluation of the EB type of a patient, the severity as well as the visibility of EB should be included in further investigations as relevant patient reported measures.

Similarly to the degree of severity and visibility and due to the chronic nature of the disease, also the EB phase should be considered as relevant in the assessment of the health status of a patient and its effects on their relatives. Again, the proportion of patients in an acute phase was higher in the group of relatives. Patients in an acute phase had more medical symptoms, they and their relatives were more burdened and less satisfied with their situation, had fewer resources available and rated themselves as less resilient. Relatives of EB patients in an acute phase additionally reported less social support. One possible explanation might be that due to the high level of stress in an acute phase, patients and relatives experience and hence rate their own resilience as lower and available and existing external support might feel less accessible. Patients with a restricted mobility due to EB symptoms also stated that they had fewer resources available. Again, patients might be less aware of the additional support.

A central theme of the interview study was the financial burden associated with EB. The results of the online survey showed that the participants who were burdened and not satisfied by their financial situation had fewer resources and less social support available. The reported bureaucratic procedures associated with financial support for EB patients and their families contributes to the burden and dissatisfaction with their financial situation. Wound care and pain management of more severely affected EB patients require a lot of attention [8, 49]. The additional dealing with financial issues and the potential income loss due to care necessities puts further stress on the EB patients and their relatives [50]. Only a small portion of our participants reported to get all their expenses for bandages, medicine and adequate care covered by the insurance. The patient organization DEBRA Austria bridges these shortcomings of the public care structure and offers financial support. More than half of the participants stated that they had received such financial support several times to regularly, and almost all of them are very content with it.

Concerning external support, participants received frequent and regular medical support. In contrast to that, nonmedical support was used less frequently, but the overall contentment with the received support was high, independent of the frequency. The results underline that contentment with the available medical support in Austria is high amongst EB patients and their relatives. However, as already suggested, the availability of nonmedical support should be extended, especially for less severely affected patients and their relatives. Future research should identify factors that contribute to the limited availability of non-medical support to improve accessibility and provide comprehensive support to EB families.

The findings of the online survey emphasises the huge impact of EB on the psychological well-being of patients and family members. Nearly half of the participants reported to be unhappy (41.5%), while 46.9% were burdened by their feelings. Additionally, 49.0% expressed burdens due to their worries. Unhappy or emotionally burdened participants reported, e.g., fewer resources, less satisfaction or less quality of life. Participants with a happy mood reported fewer burdens, a higher quality of life, more satisfaciction, more resources, social support and helpful practices available. This is in accordance with previous findings, linking a higher happiness to a higher quality of life [51]. Still, only 23.2% of the participants reported that they had received psychological or psychotherapeutic support several times and only 12.5% regularly, and rather participants reporting more medical symptoms and a higher burden. This indicates that patients and relatives with a high degree of severity rather feel psychologically burdened, but also rather have access to psychological support. However, the numbers above illustrate that there is a high need for psychological support for all degrees of severity [13].

It would be valuable to compare the presented data with findings from other countries. Future studies could aim at collecting data on a local level or at permitting a global perspective on quality of life with EB.

## Transfer and implications

**Table:** 

**What actions should be taken, according to the participants’ statements:**
▪ Coverage of costs for bandages, medication, medical care and 24h care through the public health system
▪ Targeted support
- in regard to the intra-family distribution of (care) responsibilities
- for career planning and job search
- for milder types
- for dealing with limited mobility
▪ More awareness about EB and its visible symptoms in the general population and in the medical field
➜ Actions or interventions for strengthening individual resources

The **financial situation** in particular can have a negative impact on those affected and their families. Only 21% of the participants reported that their access to bandages and medication is fully covered, whereas only 12% reported a fully funded access to adequate medical care. In addition, it was often mentioned in the interviews that the bureaucracy related to financial support is an "unnecessary additional burden" on top of the disease. Participants expressed the need for higher coverage of EB related costs by the public health care system and simplified reimbursement procedures, preferably in the form of automated online procedures.

„Simplify the bureaucracy for the reimbursement of remedies - this is an incredible effort and an unnecessary additional burden“ (627, pos. 2).

“Simplified application for replacement of medicines/supplements or preferably all reimbursements automated (should be possible by now in the age of digitalization)” (2683, pos. 2)

The combination of a high financial burden with a high effort family life can put a lot of additional burden on families. Families target this struggle by either relying on external professional support (e.g. 24h care) or by doing the mandatory care themselves. However, less financial support is provided for the latter option in Austria. Often it is not or only partly possible to externalise the care. Some participants expressed the wish to take over the care themselves while receiving adequate financial support (as with professional 24-hour care) in order to reduce the accumulation of multiple burdens.

“24 hour care by relatives and a job under one roof. It would be desirable if I, as a relative, could choose between a job or 24 hour care. Therefore, a basic salary for the relatives who lovingly take over the 24-hour care without financial deductions for the EB patients.” (2104, pos. 1)

“Basic income support for those affected and their relatives in the amount of a salary, as with the official 24-hour care benefit. Because 40 hours of work per week and 24 hours of care cannot be managed. Work or 24-hour care. To have enough free time for own and joint activities.” (606, pos. 3).

“Financial support to pay a relative for 24 hour care. So that I can maintain my social needs.” (603, pos. 2)

The **distribution of care** should be actively discussed. Making all tasks visible by making them explicit could help identify exuberant responsibilities of specific family members. A willingness to remain flexible and to divide, share, or take turns with tasks could benefit all family members. It is recommended to consider the involvement of external support for care tasks. Relatives retrospectively emphasised the gains from including several caregivers into wound care from early on. Families with a new-born EB baby should be made aware that involving different caregivers at an early stage can reduce the development of dependency on one main caregiver, lead to more flexibility, both on the patients’ and the relatives’ sides, and reduce burden in the long run.

“That's what we tell all parents of small children with EB, pay attention, do it immediately, make sure immediately that your child is used to daddy doing it. Or often daddy can't do it either, because daddy might be working, as it was the case for me […]. But you could also make it natural for an affected person to just have a third party, a nurse or a caregiver, to just do that as well.” (A6, pos. 13)

The extent of the various possible burdens EB can have on a family became particularly clear. The participants stated that it is important to prevent excessive stress early on and, if financially possible, to hand over some tasks like, e.g., babysitting or cleaning in order to counteract long-term overload. Parents of a child with EB should be reminded and supported to take time for themselves and their partnership on a regular basis, which has the potential to strengthen personal resources and lead to a higher quality of life in the long run.

“It was so important for me that the children were doing well and [name of the partner] and I simply forgot about myself. And at some point that doesn't work anymore. [...] We tried to spend our free time as much as possible with the children and our joint activities. But for me, the first years, I don't think I had much free time. Maybe a visit to the cinema, maybe the theatre. I would recommend to everyone that they take better care of themselves and look after themselves a bit more, have babysitters more often and get more support.” (A7, pos. 3)

**Career plans** should be made in accordance with the individual situation and might be influenced by the nature and severity of EB. Professional career counselling provided by people who have sufficient knowledge about EB would be helpful in this context.

“Work, career choice” (593, pos. 1)

Although DEBRA Austria has already made enormous efforts to raise general awareness of EB, patients express the wish to be seen as 'normal people', especially in the context of **professional opportunities**. In this sense, targeted education of potential employers aimed to clarify that people with EB, regardless of their condition, should be seen as possible employees who can make a relevant contribution in a suitable environment. In addition, employment opportunities tailored to the needs and possibilities of EB patients would be desirable.

“Job search” (1764, pos. 1)

“What doesn't exist at all is that people with a chronic illness are seen as normal, as human beings, and are also treated as such, that there are quasi tailor-made offers where they can just go to school normally, work, do an apprenticeship or training. For [name of person affected], the most important thing would be to be able to participate normally in life. But he often can't, because people say beforehand, you have this and that anyway.” (A10, pos. 3)

**Mobility** also had a significant impact on quality of life. Even though mobility is described as a major challenge of those living with EB in Austria, only 26% have seen an occupational therapist. Helpful practices in this context are often associated with additional expenses: suitable wheelchairs or personal support such as recreational care. Targeted support for limited mobility can mostly take the form of financial or organisational support. On a societal level, accessibility, especially in public transport, is the key to independence despite limited mobility.

In addition, higher visibility of EB is associated with a higher burden. Despite the high **general awareness** of the disease among the Austrian public, thanks to the great efforts of DEBRA Austria, patients and their family members still feel burdened. In the future, further awareness raising could emphasise that EB is not a transmittable disease and focus on de-stigmatising scars and bandages. On the other hand, not having a visible disease can also be burdensome. For example, some participants have expressed that their burden is not perceived because their EB is not visible. In particular, people with milder variants sometimes feel overlooked and do not find understanding in their environment for the great effort associated with the disease. Targeted education about the effects of the disease on milder and less visible forms would be useful in the future.

"I also know from other people that they too were looked down upon and not taken seriously because of the blisters. They don't know mild forms of EB." (557, pos. 4)

"Since you can't see my EB, it's sometimes hard to get friends to understand that I can't go hiking with them, for example, or that I can't wear 'nice' ball shoes." (590, pos. 1)

"The fact of having to justify the extra time / attention / care - because for outsiders (also relatives) it is not visible how much effort in care is necessary (especially if at first sight there are no big wounds, because all open areas are on mucous membranes etc...)". (627, pos. 1)

Even among medical professionals or authorities, there is still a lack of information about the limitations that the disease can bring to affected persons and their relatives, even in the so-called mild forms. In case of a reduced visibility of the disease, people with milder forms sometimes have to accept misjudgements and dismissive comments. Therefore, even in a medical context, caution is needed to ensure that milder forms are not overlooked.

"Some authorities and doctors who classify this disease as harmless must be better informed. […] Acknowledging this walking disability and not having to be told ‘they will be able to walk a few steps’ […] (e.g. when parking)” (2193, pos. 4)

Finally, there is still considerable need for improvement in the development and maintenance of resources for EB patients and their families, and for more targeted support. Interventions should be gender-specific and adapted to the individual situation of affected persons and families.

## Conclusion and outlook

The results of the mixed methods study show that the impact of EB on the psychosocial well-being of EB patients and their relatives in Austria varies depending on the current health status, the physical and psychosocial burden, the visibility, the severity, the gender, the EB type and the financial burden. Since the perceived social support, resilience, helpful practices as well as personal and social resources significantly influence the satisfaction and/or quality of life, an essential next step should focus on coverage of EB-related costs through the public health system, on targeted support for specific subpopulations and topics, and on further raising awareness about EB in the general population and in the medical field. The aim should be to raise the level of well-being of EB patients and relatives by strengthening resources on a structural and on an individual level.

### Supplementary Information

**Supplementary Material 1.** 

## Data Availability

The data generated during and/or analysed during the current study are not publicly available nor are they available on request due to the rarity of EB, which limits anonymity even with pseudonymisation or exclusion of personal data.

## Footnotes

1 For a description of the patient organisation DEBRA Austria and their services, please visit https://www.debra-austria.org.
