# Supplementary material for: Facing the complex challenges of people with epidermolysis bullosa in Austria: a mixed methods study on burdens and helpful practices
Source: Orphanet J Rare Dis. 2024 May 21;19:211. doi: 10.1186/s13023-024-03163-4 (PMC11110252; doi:10.1186/s13023-024-03163-4)
Supplement: Supplementary file 1 — Supplementary Material 1. [file 13023_2024_3163_MOESM1_ESM.pdf]

Additional Material

Table A1. Group comparison matrix of all categories' medians

	Current health ↓	Quality of life ↓		Burdens ↑	Satisfaction ↑	Resources ↑	Helpful practices ↑	Resilience ↑	Social support ↑	General satisfaction ↑
	iscorEB	For patients: QOLEB	For relatives: EB-BoD	ResILL-EB Burden scale	ResILL-EB Satisfaction scale	ResILL-EB Resources scale	ResILL-EB Helpful practices	BRS	F-SozU	SWLS
**Gender**	p=.014*[1]	p=.029	p=.008	p=.025*[1]	*No significant differences*	p=.001*[2]	p=.008	*No significant differences*	p=.005	p=.045*[2]
Female	MD=2.53	MD=1.71	MD=0.89	MD=3.98		MD=2.60	MD=2.00		MD=4.67	MD=5.80
Male	MD=0.93	MD=1.29	MD=1.94	MD=1.38		MD=2.00	MD=1.83		MD=4.00	MD=5.00
**EB-Form**	p<.001	*No significant differences*	*No significant differences*	p=.004	p=.001	p=.006	*No significant differences*	*No significant differences*	*No significant differences*	p=.046
EBS	MD=1.20			MD=2.00	MD=8.67	MD=2.73				MD=5.80
DEB	MD=2.53			MD=3.65	MD=6.94	MD=2.35				MD=5.30
JEB	MD=3.27			MD=5.43	MD=5.00	MD=2.00				MD=3.80
**Severity**	p<.001	p=-022	p=.005	p=.001	p=.025	*No significant differences*	*No significant differences*	*No significant differences*	*No significant differences*	*No significant differences*
Mild	MD=1.20	MD=1.35	MD=0.64	MD=2.20	MD=8.44					
Moderate/severe	MD=2.83	MD=1.71	MD=1.94	MD=4.45	MD=6.78					
**Visibility**	p=.034	*No significant differences*	*No significant differences*	p=.011	*No significant differences*	*No significant differences*	*No significant differences*	*No significant differences*	*No significant differences*	*No significant differences*
Hardly	MD=1.20			MD=1.15						
Somewhat	MD=2.53			MD=2.80						
Very	MD=2.53			MD=4.50						
**Acute EB phase**	p=.005	p=.023	*No significant differences*	p=.014	p=.001	p=.033	*No significant differences*	p=.033	p=.030*[2]	*No significant differences*
Yes	MD=3.33	MD=2.09		MD=4.50	MD=5.50	MD=2.15		MD=3.00	MD=3.50	
No	MD=2.00	MD=1.53		MD=2.45	MD=7.89	MD=2.60		MD=3.75	MD=4.58	
**Limited mobility**	p=.006	*No significant differences*	*No significant differences*	*No significant differences*	*No significant differences*	p=.046	*No significant differences*	*No significant differences*	*No significant differences*	*No significant differences*
Yes	MD=2.53					MD=2.15				
no	MD=1.60					MD=2.45				
**Overall support**	p<.001	*No significant differences*	*No significant differences*	p=.021	*No significant differences*	*No significant differences*	*No significant differences*	*No significant differences*	*No significant differences*	*No significant differences*
Frequently	MD=3.73			MD=5.05						
No support	MD=2.00			MD=2.35						
**Psychologic/ therapeutic support**	p=.003	*No significant differences*	*No significant differences*	p=.035	*No significant differences*	*No significant differences*	p=.037	*No significant differences*	*No significant differences*	*No significant differences*
Frequently:	MD=2.93			MD=4.50			MD=2.03			
Not/once	MD= 2.00			MD=2.30			MD=1.82			
**Financial burden**	p<.001	*No significant differences*	*No significant differences*	*No significant differences*	p=.014	p<.001	*No significant differences*	*No significant differences*	p=.009	p=.001
Burdened	MD=3.40				MD=6.67	MD=2.00			MD=4.00	MD=4.00
Not/rather not	MD=1.73				MD=7.67	MD=2.70			MD= 4.58	MD=5.80
**Financial satisfaction**	p<.001	*No significant differences*	*No significant differences*	p=.004	*No significant differences*	p=.002	*No significant differences*	*No significant differences*	p=.012	p=.004
Satisfied	MD=1.60			MD=2.20		MD=2.70			MD=4.67	MD=5.80
Not/rather not	MD=3.20			MD=4.50		MD=2.05			MD=3.83	MD=3.80
**Mood**	*No significant differences*	*No significant differences*	*No significant differences*	p=.007	p=.002	p=.008	p=.038	*No significant differences*	p=.037	*No significant differences*
Happy				MD=2.20	MD=7.56	MD=2.60	MD=2.00		MD=4.50	
Unhappy				MD=4.50	MD=6.00	MD=2.05	MD=1.76		MD=4.00	
**Burden due to feelings**	p=.004	*No significant differences*	*No significant differences*	*No significant differences*	p=.005	p=.041	*No significant differences*	*No significant differences*	*No significant differences*	*No significant differences*
Burdened	MD=2.53				MD=6.78	MD=2.15				
Not/hardly	MD=1.67				MD=8.11	MD=2.50				
**Burden due to worries and fears**	p<.001	*No significant differences*	*No significant differences*	*No significant differences*	p<.001	p<.001	p=.009	p=.017	p=.033	p=.002
Burdened	MD=3.40				MD=6.56	MD=2.00	MD=1.76	MD=3.33	MD=4.00	MD=4.40
Not/hardly	MD=1.60				MD=8.44	MD=2.70	MD=2.03	MD=3.83	MD=4.50	MD=5.80

**1 only for patients significant differences, *2 only for relatives significant differences, ↑= Higher values indicate higher expression in the targeted construct, ↓= Lower values indicate higher expression in the targeted construct*

	Current health ↓	Quality of life ↓		Burdens ↑	Satisfaction with EB ↑	Resources ↑	Helpful strategies ↑	Resilience ↑	Social support ↑	Overall satisfaction ↑
	iscorEB	For patients: QOLEB	For relatives: EB-BoD	ResILL-EB Burden scale	ResILL-EB Satisfaction scale	ResILL-EB Resources scale	ResILL-EB Helpful strategies	BRS	F-SozU	SWLS
**Gender**	p^{*1}=.014	*p*=.029	*p*=.008	*p*=.025^{*1}	-	p^{*2}=.001^{*2}	*p*=.008	-	*p*=.005	p^{*2}=.045
Female	**MD=2.53**	**MD=1.71**	MD=0.89	**MD=4.98**		**MD=2.60**	**MD=2.00**		**MD=4.67**	**MD=5.80**
Male	MD=0.93	MD=1.29	**MD=1.94**	MD=2.38		MD=2.00	MD=1.83		MD=4.00	MD=5.00
**EB-Form**	*p*<.001	-	-	*p*=.004	*p*=.001	*p*=.006	-	-	-	*p*=.046
EBS	MD=1.20			MD=3.00	**MD=9.67**	**MD=2.73**				**MD=5.80**
DEB	MD=2.53			MD=4.65	MD=7.94	MD=2.35				MD=5.30
JEB	**MD=3.27**			**MD=6.43**	MD=6.00	MD=2.00				MD=3.80
**Severity**	*p*<.001	*p*=-022	*p*=.005	*p*=.001	*p*=.025	-	-	-	-	-
Mild	MD=1.20	MD=1.35	MD=0.64	MD=3.20	**MD=9.44**					
Moderate/severe	**MD=2.83**	**MD=1.71**	**MD=1.94**	**MD=5.45**	MD=7.78					
**Visibility**	*p*=.034	-	-	*p*=.011	-	-	-	-	-	-
Hardly	MD=1.20			MD=2.15						
Somewhat	**MD=2.53**			MD=3.80						
Very	**MD=2.53**			**MD=5.50**						
**Acute EB phase**	*p*=.005	*p*=.023	-	*p*=.014	*p*=.001	*p*=.033	-	*p*=.033	p^{*2}=.030	-
Yes	**MD=3.33**	**MD=2.09**		**MD=5.50**	MD=6.50	MD=2.15		MD=3.00	MD=3.50	
No	MD=2.00	MD=1.53		MD=3.45	**MD=8.89**	**MD=2.60**		**MD=3.75**	**MD=4.58**	
**Limited mobility**	*p*=.006	-	-	-	-	*p*=.046	-	-	-	-
Yes	**MD=2.53**					MD=2.15				
no	MD=1.60					**MD=2.45**				
**Overall support**	*p*<.001	-	-	*p*=.021	-	-	-	-	-	-
Frequently	**MD=3.73**			**MD=6.05**						
No support	MD=2.00			MD=3.35						
**Psychologic/ therapeutic support**	*p*=.003	-	-	*p*=.035	-	-	*p*=.037	-	-	-
Frequently:	**MD=2.93**			**MD=5.50**			**MD=2.03**			
Not/once	MD= 2.00			MD=3.30			MD=1.82			
**Financial burden**	*p*<.001	-	-	-	*p*=.014	*p*<.001	-	-	*p*=.009	*p*=.001
Burdened	**MD=3.40**				MD=7.67	MD=2.00			MD=4.00	MD=4.00
Not/rather not	MD=1.73				**MD=8.67**	**MD=2.70**			**MD= 4.58**	**MD=5.80**
**Financial satisfaction**	*p*<.001	-	-	*p*=.004		*p*=.002	-	-	*p*=.012	*p*=.004
Satisfied	MD=1.60			MD=3.20		**MD=2.70**			**MD=4.67**	**MD=5.80**
Not/rather not	**MD=3.20**			**MD=5.50**		MD=2.05			MD=3.83	MD=3.80
**Mood**	-	-	-	*p*=.007	*p*=.002	*p*=.008	*p*=.038	-	*p*=.037	-
Happy				MD=3.20	**MD=8.56**	**MD=2.60**	**MD=2.00**		**MD=4.50**	
Unhappy				**MD=5.50**	MD=7.00	MD=2.05	MD=1.76		MD=4.00	
**Burden due to feelings**	*p*=.004	-	-	-	*p*=.005	*p*=.041	-	-	-	-
Burdened	**MD=2.53**				MD=7.78	MD=2.15				
Not/hardly	MD=1.67				**MD=9.11**	**MD=2.50**				
**Burden due to worries and fears**	*p*<.001	-	-	-	*p*<.001	*p*<.001	*p*=.009	*p*=.017	*p*=.033	*p*=.002
Burdened	**MD=3.40**				MD=7.56	MD=2.00	MD=1.76	MD=3.33	MD=4.00	MD=4.40
Not/hardly	MD=1.60				**MD=9.44**	**MD=2.70**	**MD=2.03**	**MD=3.83**	**MD=4.50**	**MD=5.80**

**[1] only for patients significant differences, *[2] only for relatives significant differences*

	Current health ↓	Quality of life ↓		Burdens ↑	Satisfaction with EB ↑	Resources ↑	Helpful strategies ↑	Resilience ↑	Social support ↑	Overall satisfaction ↑
	iscorEB	For patients: QOLEB	For relatives: EB-BoD	ResILL-EB Burden scale	ResILL-EB Satisfaction scale	ResILL-EB Resources scale	ResILL-EB Helpful strategies	BRS	F-SozU	SWLS
**Gender**	p^{*1}=.014	p=.029	p=.008	p=.025^{*1}	-	p^{*2}=.001^{*2}	p=.008	-	p=.005	p^{*2}=.045
Female	**MD=2.53**	**MD=1.71**	MD=0.89	**MD=4.98**		**MD=2.60**	**MD=2.00**		**MD=4.67**	**MD=5.80**
Male	MD=0.93	MD=1.29	**MD=1.94**	MD=2.38		MD=2.00	MD=1.83		MD=4.00	MD=5.00
**EB-Form**	p<.001	-	-	p=.004	p=.001	p=.006	-	-	-	p=.046
EBS	MD=1.20			MD=3.00	**MD=9.67**	**MD=2.73**				**MD=5.80**
DEB	MD=2.53			MD=4.65	MD=7.94	MD=2.35				MD=5.30
JEB	**MD=3.27**			**MD=6.43**	MD=6.00	MD=2.00				MD=3.80
**Severity**	p<.001	p=-022	p=.005	p=.001	p=.025	-	-	-	-	-
Mild	MD=1.20	MD=1.35	MD=0.64	MD=3.20	**MD=9.44**					
Moderate/severe	**MD=2.83**	**MD=1.71**	**MD=1.94**	**MD=5.45**	MD=7.78					
**Visibility**	p=.034	-	-	p=.011	-	-	-	-	-	-
Hardly	MD=1.20			MD=2.15						
Somewhat	**MD=2.53**			MD=3.80						
Very	**MD=2.53**			**MD=5.50**						
**Acute EB phase**	p=.005	p=.023	-	p=.014	p=.001	p=.033	-	p=.033	p^{*2}=.030	-
Yes	**MD=3.33**	**MD=2.09**		**MD=5.50**	MD=6.50	MD=2.15		MD=3.00	MD=3.50	
No	MD=2.00	MD=1.53		MD=3.45	**MD=8.89**	**MD=2.60**		**MD=3.75**	**MD=4.58**	
**Limited mobility**	p=.006	-	-	-	-	p=.046	-	-	-	-
Yes	**MD=2.53**					MD=2.15				
no	MD=1.60					**MD=2.45**				
**Overall support**	p<.001	-	-	p=.021	-	-	-	-	-	-
Frequently	**MD=3.73**			**MD=6.05**						
No support	MD=2.00			MD=3.35						
**Psychologic/ therapeutic support**	p=.003	-	-	p=.035	-	-	p=.037	-	-	-
Frequently:	**MD=2.93**			**MD=5.50**			**MD=2.03**			
Not/once	MD= 2.00			MD=3.30			MD=1.82			
**Financial burden**	p<.001	-	-	-	p=.014	p<.001	-	-	p=.009	p=.001
Burdened	**MD=3.40**				MD=7.67	MD=2.00			MD=4.00	MD=4.00
Not/rather not	MD=1.73				**MD=8.67**	**MD=2.70**			**MD= 4.58**	**MD=5.80**
**Financial satisfaction**	p<.001	-	-	p=.004		p=.002	-	-	p=.012	p=.004
Satisfied	MD=1.60			MD=3.20		**MD=2.70**			**MD=4.67**	**MD=5.80**
Not/rather not	**MD=3.20**			**MD=5.50**		MD=2.05			MD=3.83	MD=3.80
**Mood**	-	-	-	p=.007	p=.002	p=.008	p=.038	-	p=.037	-
Happy				MD=3.20	**MD=8.56**	**MD=2.60**	**MD=2.00**		**MD=4.50**	
Unhappy				**MD=5.50**	MD=7.00	MD=2.05	MD=1.76		MD=4.00	
**Burden due to feelings**	p=.004	-	-	-	p=.005	p=.041	-	-	-	-
Burdened	**MD=2.53**				MD=7.78	MD=2.15				
Not/hardly	MD=1.67				**MD=9.11**	**MD=2.50**				
**Burden due to worries and fears**	p<.001	-	-	-	p<.001	p<.001	p=.009	p=.017	p=.033	p=.002
Burdened	**MD=3.40**				MD=7.56	MD=2.00	MD=1.76	MD=3.33	MD=4.00	MD=4.40
Not/hardly	MD=1.60				**MD=9.44**	**MD=2.70**	**MD=2.03**	**MD=3.83**	**MD=4.50**	**MD=5.80**

^{*1} only for patients significant Differences, ^{*2} only for relatives significant differences
